# Assessment of Ablation Therapy in Pancreatic Cancer: The Radiologist’s Challenge

**DOI:** 10.3389/fonc.2020.560952

**Published:** 2020-11-27

**Authors:** Vincenza Granata, Roberta Grassi, Roberta Fusco, Sergio Venanzio Setola, Raffaele Palaia, Andrea Belli, Vittorio Miele, Luca Brunese, Roberto Grassi, Antonella Petrillo, Francesco Izzo

**Affiliations:** ^1^ Radiology Division, Istituto Nazionale Tumori, IRCCS, Fondazione G. Pascale, Naples, Italy; ^2^ Radiology Division, Universita’ Degli Studi Della Campania Luigi Vanvitelli, Naples, Italy; ^3^ Hepatobiliary Surgical Oncology Division, Istituto Nazionale Tumori, IRCCS, Fondazione G. Pascale, Naples, Italy; ^4^ Department of Radiology, Careggi University Hospital, Florence, Italy; ^5^ Department of Medicine and Health Sciences “V. Tiberio,” University of Molise, Campobasso, Italy

**Keywords:** pancreatic cancer, ablation treatment, computed tomography, magnetic resonance imaging, functional imaging

## Abstract

This article provides an overview of imaging assessment of ablated pancreatic cancer. Only studies reporting radiological assessment on pancreatic ablated cancer were retained. We found 16 clinical studies that satisfied the inclusion criteria. Radiofrequency ablation and irreversible electroporation have become established treatment modalities because of their efficacy, low complication rates, and availability. Microwave Ablation (MWA) has several advantages over radiofrequency ablation (RFA), which may make it more attractive to treat pancreatic cancer. Electrochemotherapy (ECT) is a very interesting emerging technique, characterized by low complication rate and safety profile. According to the literature, the assessment of the effectiveness of ablative therapies is difficult by means of the Response Evaluation Criteria in Solid Tumors (RECIST) criteria that are not suitable to evaluate the treatment response considering that are related to technique used, the timing of reassessment, and the imaging procedure being used to evaluate the efficacy. RFA causes various appearances on imaging in the ablated zone, correlating to the different effects, such as interstitial edema, hemorrhage, carbonization, necrosis, and fibrosis. Irreversible electroporation (IRE) causes the creation of pores within the cell membrane causing cell death. Experimental studies showed that Diffusion Weigthed Imaging (DWI) extracted parameters could be used to detect therapy effects. No data about functional assessment post MWA is available in literature. Morphologic data extracted by Computed Tomography (CT) or Magnetic Resonance Imaging (MRI) do not allow to differentiate partial, complete, or incomplete response after ECT conversely to functional parameters, obtained with Position Emission Tomography (PET), MRI, and CT.

## Introduction

Oncology disease is the second principal cause of death in both men and women. Incidence continues to increase for pancreatic cancer, with an estimated death rate of 81.7% among new cases of 2020 and a 5-year relative survival rate of the 9% (1). The decision regarding resectability status of pancreatic cancer should be made by the multidisciplinary meetings consensus following the acquisition of pancreatic imaging including complete staging. In fact, most patients had locally advanced or metastatic disease at diagnosis, and systemic chemotherapy is usually the main treatment (2–6). Most patients experience relapse after treatment. Furthermore, the “cure rate” for this disease is only 9%, and without treatment, the median survival of patients with metastatic disease is only 3 months. First-line treatment regimens consists of FOLFIRINOX and gemcitabine/albumin-bound nab-paclitaxel, and for patients with BRCA1/2 and PALB2 mutations, gemcitabine/cisplatin. Compared with nab-paclitaxel/gemcitabine, FOLFIRINOX may be associated with a somewhat better response rate and progression-free and overall survival (OS), but it is a difficult regimen that is best reserved for fit patients (3). Despite the latest introduction of new treatment schemes, chemotherapy in advanced pancreatic cancers still correlates to an unfortunate long-term survival and considerable ad interim complications (6, 7). The resectability assessment of Locally Advanced Pancreatic Cancer (LAPC) after neoadjuvant therapy is still challenging. In dedicated cancer centers, patients with persistent LAPC after chemotherapy should be subjected to local treatment if they are in good clinical condition (WHO Performance Status 0–1) and according to Response Evaluation Criteria in Solid Tumors (RECIST) in stable disease after 2–4 months chemotherapy. However, randomized trials to assess the ablative therapies additional value to chemotherapy-alone are lacking and currently there are no completed trials comparing multiple ablative approaches (8). Additionally, there is increasing suggestion that local ablative therapies can induce a systemic anti-tumor response (8).

Today ablative therapies should be used as consolidative treatment in stable disease (9). Assessment after ablative treatment is complicated and is related to the type of treatment used (10–15). Radiofrequency ablation (RFA) and microwave ablation (MWA) are hyperthermic tools that use energy to heat the target area to at least 60°C (16, 17). Although the technological features of RFA and MWA are comparable, the differences occur from the physical phenomenon used to create heat. In fact, RFA is based on thermocoagulation necrosis, while MWA causes cellular death thanks to dielectric heating (16, 17).

The cell membrane permeability changes induced by the application of an external electric field is called electroporation. Electroporation can be applied in either an irreversible (IRE) (14, 18–28) or a reversible manner (11–13, 29, 30), depending on the electrical field strength and duration. IRE is based on alteration of the transmembrane potential, causing the disruption of the lipid bilayer by the creation of small pores (“nanopores”), thus driving the cells toward apoptosis (23). Reversible electroporation can be used in combination with administration of a chemotherapeutic drug (ECT) or also gene therapy and vaccination (Electrogenetransfer, EGT). ECT is based on the electroporation of cells and the associated administration of low doses of a chemotherapeutic agent, especially bleomycin (BLM). An external electrical field is applied to the cell membrane inducing a transient and reversible orientation of its polar molecules, consequently there is an increase in cell permeability with a higher dose of chemotherapeutic agent that can penetrate (11). ECT determines a direct toxic phenomenon and an anti-vascular effect. “This so called ‘vascular lock’ effect retains the chemotherapeutic agent in the treatment area thereby increasing the treatment effect further” (31). “Furthermore, the type of cell death that is mediated is dependent on the number of intracellular BLM molecules. A few hundred to few thousand molecules lead to a slow mitotic cell death and more internalized molecules lead to a faster pseudoapoptotic cell death” (32).

Several therapies both thermal and non-thermal have the ability to stimulate anti-tumor immunity. The immune-modulatory response evidence is currently the strongest related to radiotherapy, although data is accumulating for high-intensity focused ultrasound, radiofrequency ablation, reversible and irreversible electroporation (33–35).

RECIST are inappropriate to assess locoregional therapies, since existing morphologic response criteria do not offer the sufficient data to assess the efficacy of treatment. Therefore, establishment of response evaluation criteria devoted to ablation therapies is needed in clinical practice, as well as in clinical trials. According to García-Figueiras et al. functional features could predict treatment success before size changes become evident (15).

Our purpose is reporting an overview and update of imaging techniques in the response assessment to ablative therapies in pancreatic cancer.

## Methods

This overview is the result of a self-study without protocol and registration number.

### Search Criterion

We assessed several electronic databases: PubMed (US National Library of Medicine, http://www.ncbi.nlm.nih.gov/pubmed), Scopus (Elsevier, http://www.scopus.com/), Web of Science (Thomson Reuters, http://apps.webofknowledge.com/), and Google Scholar (https://scholar.goo-gle.it/). The following search criteria have been used: “Pancreatic Cancer” AND “Ablative Therapies” AND “Imaging Assessment", “Pancreatic Cancer” AND “RFA” AND “Imaging Assessment, “Pancreatic Cancer” AND “MWA” AND “Imaging Assessment, “Pancreatic Cancer” AND “IRE” AND “Imaging Assessment, “Pancreatic Cancer” AND “ECT” AND “Imaging Assessment.” According to our personal decision to assess functional imaging in evaluating ablation treatment, and since only in the last 10 years these diagnostic tool have reached their applicability, the search covered the years from January 2010 to May 2020. Moreover, the references of the found papers were evaluated for papers not indexed in the electronic database. We analyzed all titles and abstracts. The inclusion criteria was: clinical study evaluating radiological assessment of pancreatic cancer after ablative therapies. Articles published in the English language from January 2010 to May 2020 were included. Exclusion criteria were studies with no sufficient reported data, case report, review or editorial letter.

## Results

**Figure 1:**
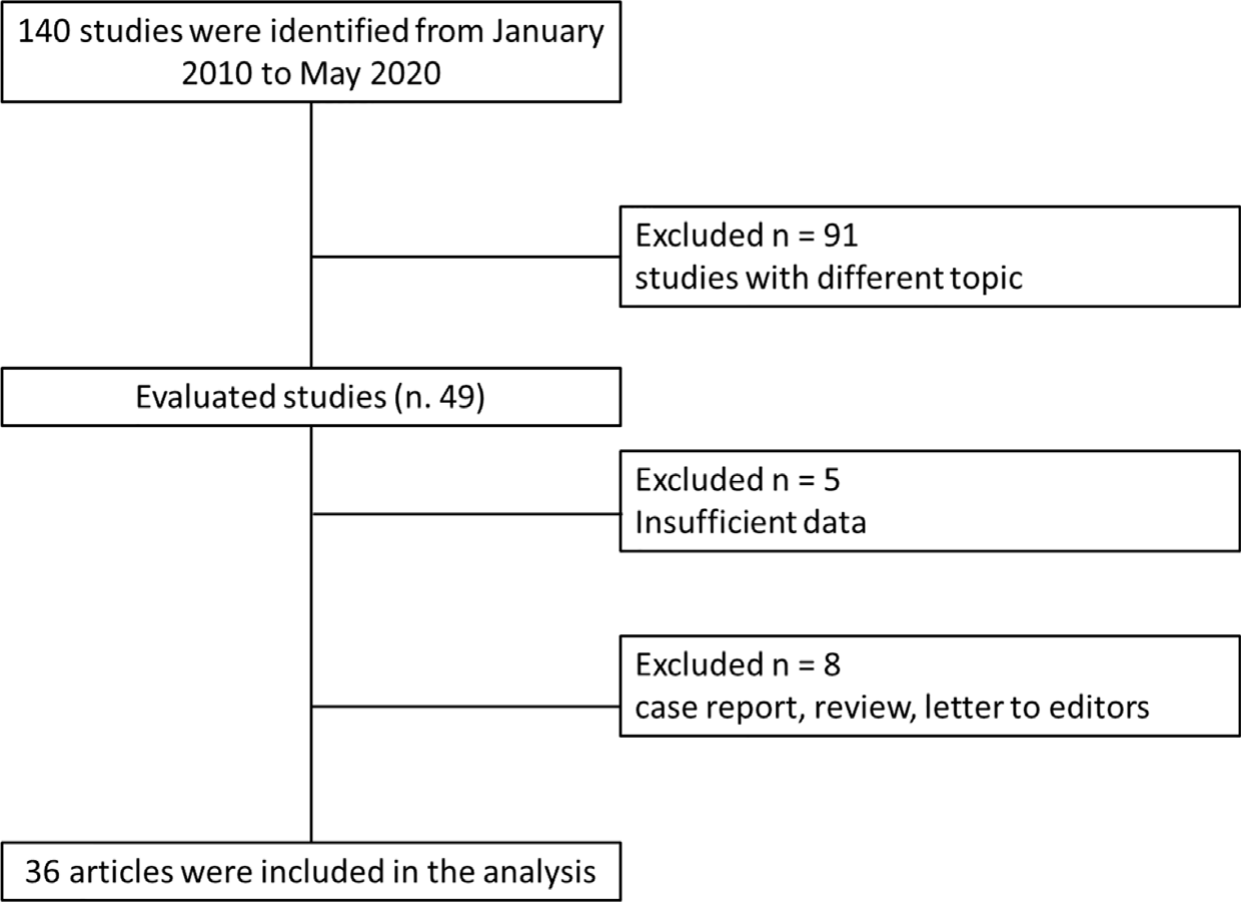
Included and excluded studies in systematic review.

We recognized 140 studies that assessed ablation treatment in pancreatic cancer from January 2010 to May 2020. Ninety-one studies have different topic in respect to the radiological assessment; 5 did not have sufficient data and 8 are case report, review, or letter to editors; so 36 articles were included at the end (**Figure 1**). We included 18 papers for RFA, 3 paper for MWA, 11 paper for IRE, and 4 paper for ECT. **Table 1** reports the mean value and the range of overall survival and the mean value of major complication rates, minor complication rates, mortality rate, and imaging analysis in pancreatic cancer treated with ablation therapies. For IRE and ECT we reported the data of the researches that have assessed significant study population, while for RFA and MWA less patients have been treated so we reported mean value considering each included study.

**Table 1:** Mean value and the range of overall survival and the mean value of major complication rates, minor complication rates, mortality rate, and imaging analysis in pancreatic cancer treated with ablation therapies.

Technique	Authors	Overall Survival (OS)	Major Complication Rates	MinorComplicationRates	MortalityRates	Imaging modality used and radiological response criteria used
**RFA**	Paiella et al. (9) D’Onofrio et al. (36) Giardino et al. (37) Hadjicostas et al. (38) Ikuta et al. (39) Kallis et al. (40) Lakhtakia (41) Pai (42) Rossi (43) Song et al. (44) Spiliotis et al. (45) Varshney et al. (46) Waung (47) Zou (48) Saccomandi (49) Zou et al. (50) Giardino (51)	23 months (mean value) 9-30 months (range)	1.9% (mean value)	20.2% (mean value)	0.7% (mean value)	CT (no functional data available) No data on MRI
**MWA**	Carrafiello et al. (52) Ierardi et al. (51) Vogl et al. (53)	–	8.5% (mean value)	8.6% (mean value)	No data reported	CT (no functional data available) No data on MRI
**IRE**	Martin et al. (20, 21) Kwon et al. (22) Lambert et al. (24) Yan et al. (25) Zhang et al. (26) Scheffer et al. (27) Narayanan et al. (54) Weiss et al. (55) Rombouts et al. (56) Scheffer et al. (57)	24.9 months (mean value) 4.9–85 months (range) [Martin et al. (20)]	1.5% Scheffer 2014 (27)	15% (open approach) 29% (percutaneus approach) Martin et al. (20) Rombouts et al. (56) Scheffer et al. (57)	2% Martin et al. (20) and Rombouts et al. (56)	CT- MRI (functional data available)
**ECT**	Granata et al. (11–13) Ongoing study by Granata et al. (58)	11.5 months (mean value)	0%	23.1% (mean value)	4.2% (mean value)	CT-MRI (functional data available)

### Radiofrequency Ablation

During RFA, the zone of active tissue heating is restricted to a few millimeters around the active needle, with the remainder of the ablation zone being heated *via* thermal conduction (16). The treatment effectiveness is related to the target size, with the best result for lesions with a size smaller than 3.5 cm (16, 17). Also, some structural characteristics of biological tissues, such as electrical and thermal conductivity, dielectric permittivity, and blood perfusion rate, have effect on the growth of ablation area. The coagulation necrosis extent is linked to the energy deposited correlated to the hepatic blood flow that with its cooling properties affected tumor ablation; this phenomenon is commonly termed “heat sink effect” (20). The heat-sink effect limits the all thermal ablation method’s effectiveness since the combined effect of electrical and thermal sink increases the incomplete necrosis risk (17–20, 59).

Today the most application of RFA on pancreatic cancer is the treatment of patients with stage-III, in case of no further systemic therapies response. However, some studies included also stage-IV patients (9). At the best of our knowledge, 18 papers assessed RFA in clinical setting (**Table 1**) (9, 36–51). In most patients RFA was reserved to stages III–IV, and in lower stage in 22 unfit-for-surgery patients. RFA was performed in 158/279 (56.6%) head lesions, in 68/279 (24.4%) body-tail lesions, and in 2 uncinate process lesions. Computed Tomography (CT) scan was the diagnostic tool mostly used to assess the treatment. Fifty-two complications were reported (**Table 1**). The most frequent were pancreatic fistula (12 cases), portal thrombosis (10 cases), and pancreatitis (8 cases). In three patients was reported duodenal injury, and in two patients abdominal bleeding. Two deaths were registered due to hepatic failure (49). In a recent review Paiella et al. reported a good oncological outcome obtained with the use of RFA on pancreatic cancers with a median OS of 30 months for patients treated with RFA, median OS of 25.6 months in the group treated RFA plus systemic therapy (**Table 1**) (9).

Recently, RFA is used as an upfront option, justified on the basis of an immunological antitumoral stimulation (50, 60).

### Microwave Ablation

MWA determines a larger zone of active heating (up to 2 cm surrounding the antenna) obtaining more uniform necrosis of the lesion. MWA benefits compared to RFA are: lesion size can be larger for larger area of necrosis determined by MWA; the treatment time is shorter (16). Carrafiello et al. (52) assessed MWA in 10 patients in stage IV, with lesion located in the head of the pancreas (**Table 1**). During the follow-up (mean time 9.2 months, range 3–16 months), the major complications rate was 30% (3 patients). Two patients developed pancreatitis and one patient pseudoaneurysm of the gastroduodenal artery. CT scan was performed up to 15 months after the treatment (**Table 1**). No patients showed a complete response. At 1 month follow-up there were found 1 progressive disease (PD), 1 partial response (PR), and 8 stable diseases (SD). Ierardi et al. (51) assessed feasibility and safety of MWA in LAPC using a new technology of MW with high power (100 W) and frequency of 2,450 MH. They treated five patients with pancreatic head cancer. Follow-up was performed by CT after 1, 3, 6, and, when possible, 12 months. The treatment was feasible in all patients (100%), observing no major complications. Minor complications resolved during the hospital stay (4 days) (**Table 1**). An improvement in Quality of Life (QoL) was observed in all patients (51). Vogl et al. (53) treated 20 pancreatic cancer patients. Seventeen lesions (77.3%) of pancreatic head cancer and 5 (22.7%) of body-tail. The efficacy reported was 100%, without major complications. Minor complications were found in 2 patients (9.1%) (severe local pain correlated to the treatment). PD was documented in one case (10%) of the 10/22 accessible 3-month follow-up MR examinations (**Table 1**).

Altogether, MWA shows promising results, however, it needs further data to improve the knowledge about the efficacy, the safety, and the oncological outcome.

### Irreversible Electroporation

IRE induces an electric field across cells in order to alter the cellular transmembrane potential. When a sufficiently high voltage is reached, the cell membrane phospholipid bilayer structure is disrupted, inducing cell apoptosis. The evidence suggests that IRE “leaves supporting tissue largely unaffected, preserving the structure of large blood vessels and bile ducts” (16, 17, 19). Since IRE efficacy is linked to electrical energy delivered; therefore its efficacy is not influenced by the heat-sink effect (16, 17, 19). This suggests safer and more effective ablation of neoplasms adjacent to large vessels or fragile structures (9–18, 20–22, 24–27, 54–56, 59).

Considering this, IRE preserves surrounding tissues and protect the vessels; this characteristic would be an essential feature when the lesion encases the major peripancreatic vessels, in which the use of thermal treatment could be unsafe and inefficacious (9–18, 20–22, 24–27, 54–56, 59).

Currently, IRE is used on stage-III LAPC (18, 27). Narayanan et al. reported three cases of IRE on stage IV (45). Also, several researchers reported the possibility to use IRE, as a technique to reduce R1 resections rate (20, 22, 54, 55). For IRE, two to six electrodes are typically placed around the tumor, with a maximum spacing of 2.0–2.5 cm. IRE has the disadvantage of necessity of general anesthesia. Rombouts et al., in a systematic review, reported complication rate of 13%, and a mortality of 2% (56). The complication rate increases with percutaneous approach (29 *vs* 15%) (20, 56, 57). Martin et al., assessing 200 treated patients, showed an overall rate of adverse events of 37% and a mortality rate of 2% (**Table 1**) (20). The most common complications described are pancreatitis, abdominal pain, bile leakage, pancreatic leakage, duodenal leakage, duodenal ulcer, pneumothorax, hematoma, and deep vein thrombosis (**Table 1**) (6). MR and CT were the diagnostic tool mostly used to assess the treatment. Despite the large number of studies on IRE in pancreatic cancer, only Martin et al. (20) reported an outstanding median Overall Survival (OS) of 24.9 months (range 12.4–85 months). Consequently, there is a need for a greater number of studies that evaluate efficacy in terms of oncological outcomes (**Table 1**).

### Electrochemotherapy

ECT is based on the electroporation of cells and the associated administration of low doses of chemotherapy. An external electric field to a cell induces a transient and reversible increase of cells transmembrane potential with a consequent increase of permeability (11–13, 30–32). Formation of the aqueous pores in the lipid bilayer is the widely recognized mechanism, but evidence is growing that individual membrane lipids and proteins changes also contribute at ECT cytotoxic effect (61). The increased accumulation of intracellular drug concentration has actually been shown both *in vitro* and *in vivo* (58, 62–65).

In the clinical setting few papers assessed the safety and efficacy of ECT in LAPC (11–13). Granata et al. (11) evaluated 13 patients with confirmed diagnosis of LAPC (stage III). In 53.8% (7/13) the lesion was on head and in 46.2% (6/13) the lesion was on body-tail. ECT was well tolerated with rapid resolution (4–8 days) of the abdominal pain. No serious adverse events occurred. No heart abnormalities were reported. No clinically significant hemodynamic or serum biologic changes were noted during or following ECT (**Table 1**). CT and MR were employed for the follow-up at 1, 3, 6, and 12 months. In an ongoing study, Granata et al. (58) showed that median OS was 11.5 months with range values of 73 months. At 1 month after ECT 76.0% of patients were in PR and 20.0% were in SD. Today, ECT is recommended during clinical studies in dedicated centres (11–13).

### Imaging Analysis

The precise detection and characterization of pancreatic lesion is still difficult. CT and MRI are the main used modalities to assess pancreatic lesions and CT has become the modality of choice in the preoperative setting and staging, so as in treatment planning and follow-up (5). However, approximately 11% of ductal adenocarcinomas are undetected at CT (10). Morever, the pancreatic cancer assessment after neoadjuvant therapy is particularly difficult and as suggested by White et al., CT would miscalculate the resectability, since diagnostic performance seems to be reduced after therapy. Therefore, there are not radiological criteria in order to assess treatment response correlated to histological response (66). The situation becomes more complicated when evaluating effectiveness of ablative therapies, considering that RECIST criteria were not suitable to assess the response (67).

### Dimensional Criteria

RECIST 1.1, based on the variation of largest diameter, do not allow to stratify the patients in responders or non-responders after ablation treatment, since after these therapies it is expected that there is an increase in the size of the ablated area. In fact, the primary endpoint of ablation therapy is to obtain a complete necrosis (similar to R0 resection) of liver tumors that is linked to create a safety margin of at least 10 mm round the external margin of the lesion (16, 68). Moreover, the nature of the pancreatic cancer, consisting of a more or less great quantity of cells fixed within a dense and fibrous stroma, reduce diagnostic accuracy when the treated area is measured (69). After effective therapy, it is difficult the differentiation between neoplastic cells and fibrosis and then it is difficult the evaluation with morphological criteria of therapy response. Moreover, a possible locoregional edema induced by treatment or inflammatory changes secondary to biliary drainage could be observed. Therefore, the treatment response evaluation for this cancer type is a serious challenge and the dimensional criteria are unsuitable (69).

### Perfusional Assessment

Perfusion CT (CTp) can provide images and quantitative measurements of hemodynamic parameters based on the linear relationship between CT enhancement and iodinated contrast agent concentration (10). Several studies evaluated perfusion CT parameters to characterize and to evaluate the treatment in patient affected by pancreatic cancer; these studies demonstrates that CTp is more able to differentiate the pancreatic disorder respect to density measurements alone. However, no significant differences in the perfusion parameters values were found between acute-chronic pancreatitis and pancreatic adenocarcinoma, then the differential diagnosis by CTp data remains difficult (10).

Dynamic contrast-enhanced (DCE)-MRI allows the calculation of quantitative parameters linked to tumor perfusion, vessel permeability and extracellular-extravascular space composition by the post processing with pharmacokinetic models of the changes in signal intensity over time after the paramagnetic contrast medium injection (69). DCE-MRI can be analyzed by qualitative, semi-quantitative, and quantitative methods (69). DCE-MRI accuracy in the evaluation of pancreatic cancer remains unclear, probably due to the fact that in pancreatic cancer, “poorly represented microvascular components could be clarified by vessel functional impairment often observed in tumors, and by the presence of a prominent stromal matrix that embeds vessels. In addition, activated pancreatic stellate cells yield increasing fibrous stroma in tumor central areas, compressing blood vessels, leading to changes in vascularity and perfusion” (69).

### Diffusion Weighted Imaging Assessment

The opportunity to obtain functional parameters by Diffusion Weighted Imaging (DWI) has facilitated the spread of this technique into clinical practice, increasing clinical confidence and decreasing false positives in the detection and characterization of lesions. DW data analysis can be done qualitatively and quantitatively, through the apparent diffusion coefficient (ADC) evaluation using a mono-exponential model at the signal intensity decay over the diffusion b values. DWI signal is linked to water mobility that related to tissue density (69). ADC can be used in the differentiation between benign and malignant tissue. Instead, the Intravoxel incoherent motion (IVIM) method used a more sophisticated process, a bi-exponential model to separately calculate the macroscopic mobility of water movement (contribution to diffusion), and microscopic movement of blood in capillaries (contribution of perfusion). Also IVIM parameters can be analyzed qualitatively and quantitatively (69). Moreover, according to the presence of microstructures, water molecules within biologic tissues exhibit a non-Gaussian phenomenon known as Diffusion Kurtosis Imaging (DKI) (69). Therefore, is possible the calculation of the kurtosis coefﬁcient (K) linked to the deviance of diffusion from a Gaussian approach, and the diffusion coefﬁcient (D) with the correction of non-Gaussian bias.

Since necrosis and perfusion modifications can happen before changes in size during therapy, DWI may aid as an early biomarker of treatment effectiveness (69).

Granata et al. showed that the perfusion-related factors extracted by DWI of pancreatic cancer, perfusion fraction (fp) and pseudo-diffusion coefficient (Dp) (linked to tumoral perfusion), mean diffusivity (MD) (linked to heterogeneous diffusion motion of water molecules in cells interstitial space) values are different from those found in normal pancreatic parenchyma and in peritumoral tissue; in addition these parameters showed better diagnostic performance than ADC (linked both perfusion and diffusion effects). The significantly different of perfusion-related factors value between cancer tissue and normal pancreatic parenchyma might be helpful for determining the most accurate diagnosis. Increased fp and MD values in peritumoral inflammation seem to suggest that DWI-derived parameters fit in the anticipated physiologic phenomena. These findings support the hypothesis that the kurtosis effect could have a better performance to differentiate pancreatic tumors, peritumoral inflammatory tissue, and normal pancreatic parenchyma (69).

### Radiomics

The extraction of innumerable quantitative features by biomedical images such as CT, MR, or positron emission tomography (PET) images is known as Radiomics. These features provide data on tumor phenotype as well as cancer microenvironment. The main challenge is the collection and optimal combination of different multimodal data sources in a quantitative method that provides unambiguous clinical parameters that allow in a precise and robust way the prediction of the results according to the upcoming decisions (70). The central hypothesis of radiomics is that individual quantitative voxel-based variables are more sensitively associated with various clinical endpoints than the more qualitative radiological and clinical data more commonly used today (70).

### Findings on Radiological Therapeutic Responses to Treatments

It is clear that, considering therapeutic responses to treatments, imaging data are sometimes complicated to understand because it depend on anatomic location, on the method of act of given therapy, on the morphological and functional criteria that are used for each imaging modality (15). In this setting, imaging observations depend highly on the type and method of therapy delivery, the timing of treatment, and the imaging technique being used to observe the effects.

**Figure 2:**
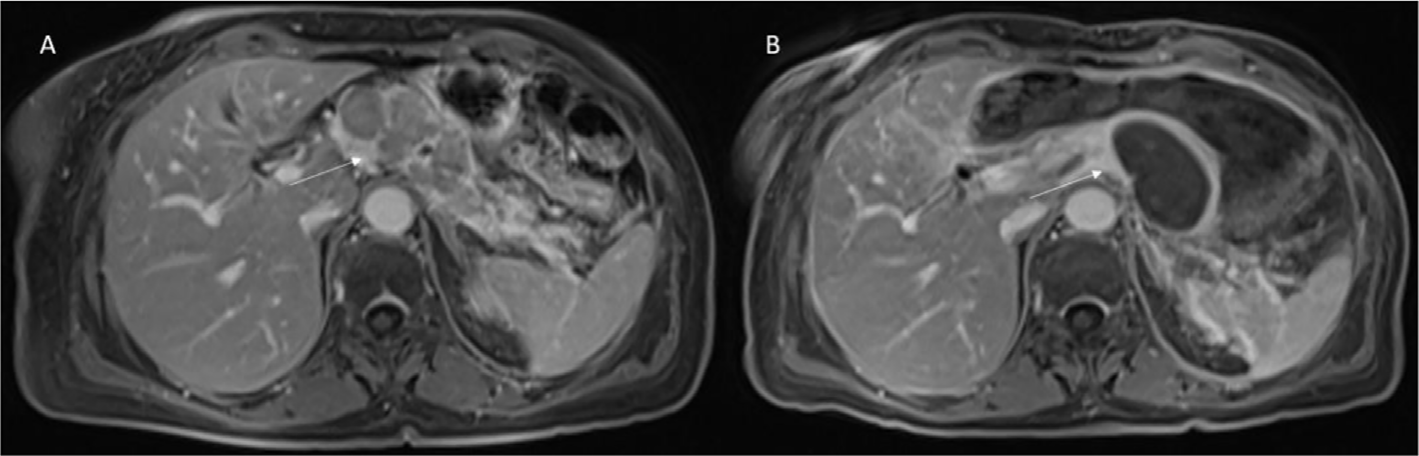
Patient 1 with Body-Tail Pancreatic Cancer. Morphological MRI assessment post-RFA treatment. In **(A)** (VIBE T1-W post-contrast sequence during portal phase in axial plane) pre-treatment evaluation of lesion (arrow). In **(B)** (VIBE T1-W post-contrast sequence during portal phase in axial plane) arrow shows ablated area. Qualitative assessment shows significant differences in SI in pre- and post-treatment sequences.

RFA causes heterogeneous appearances on imaging in the ablated areas, correlated to the therapy effects, such as interstitial edema, hemorrhage, carbonization, necrosis, and fibrosis (**Figure 2**).

Experimental studies showed that DWI could be used to detect the efficacy of IRE treatment (71, 72).

The evaluation of the treatment response in terms of lesion dimensional reduction is not appropriate because not always a positive response to treatment is linked to a size reduction; furthermore dimensional criteria do not allow the differentiation of the fibrotic tissue from the residual tumor.

Therefore, an evaluation based only on dimensional data is not appropriate to assess the efficacy of such complex treatments. However, one of the major topics evaluated by papers that we analyzed regarding the ablative techniques is the short- and long- term efficacy based on the tumor dimension reduction. Regarding RFA papers, the results on the follow-up was reported in 12 out of 18 studies for a total of 214 patients (36–41, 43, 45–48). Assessment time was between 7 and 34 months and was mainly performed by means of CT-scan and MRI (seven studies), considering only dimensional criteria (36–41, 43, 45–48). According to Paiella et al. for RFA, and in general for “thermal techniques,” the gold standard of imaging is represented by CT with a post-ablative hypointense area observed as result of the treatment (9). However, also pancreatic tumor is hypointense so that a “qualitative assessment” based only human eyes could cause misdiagnosis. A quantitative evaluation based on perfusion evaluation or metabolic analysis allows a more objective reassessment and a more correct stratification of patients in responders and non-responders to treatment (73–77).

Regarding MWA studies, the follow-up was reported in all cases. Assessment time was between 1 and 12 months, performed by CT and MRI, considering dimensional criteria.

At the best of our knowledge no papers in literature reported findings on efficacy of ablation by RFA or MWA using functional radiological approaches such as DWI, DKI, PET. On the contrary in literature are present studies about the evaluation of efficacy by IRE and ECT using several functional radiological parameters in the assessment of the treatment.

**Figure 3:**
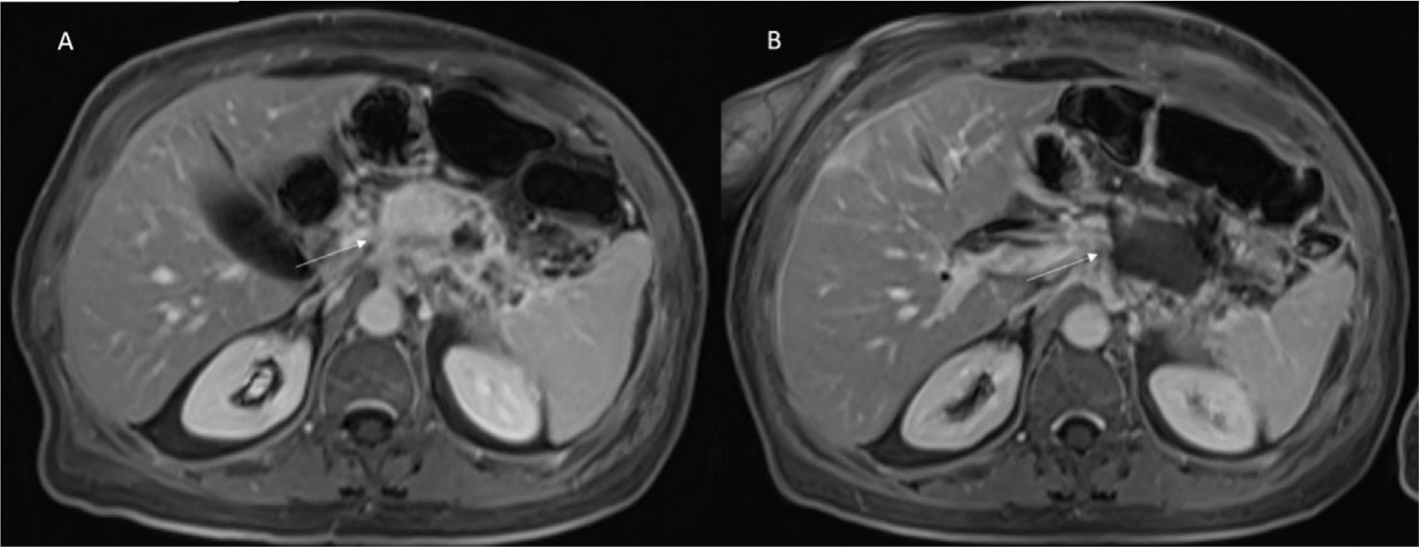
Patient 2 with Body-Tail Pancreatic Cancer. Morphological MRI assessment post-ECT treatment. In **(A)** (VIBE T1-W post-contrast sequence during portal phase in axial plane) pre-treatment evaluation of lesion (arrow). In **(B)** (VIBE T1-W post-contrast sequence during portal phase in axial plane) arrow shows ablated area. Qualitative assessment shows significant differences in SI in pre- and post-treatment sequences.

**Figure 4:**
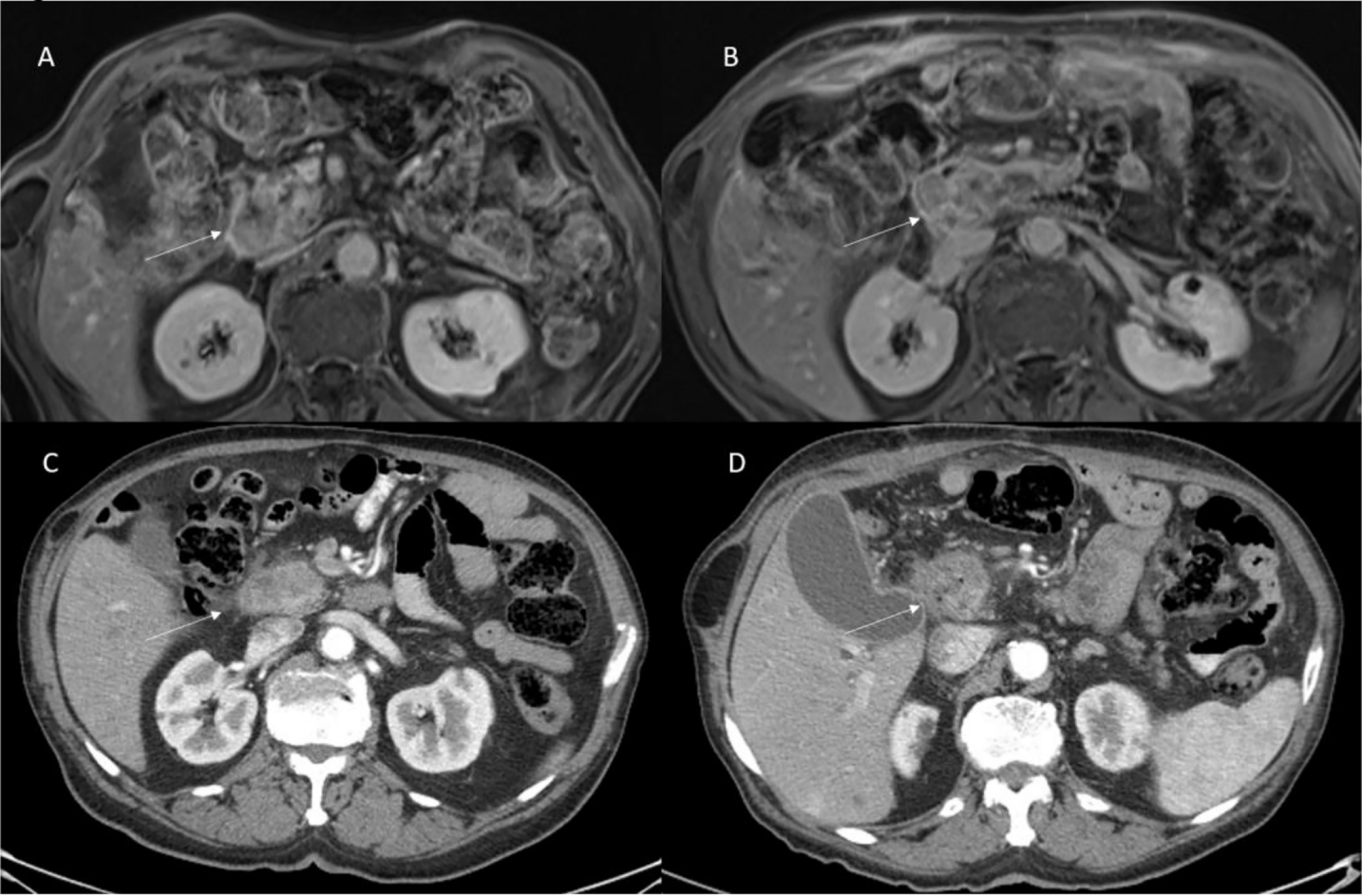
Patient 3 with head pancreatic cancer. Morphological MRI and CT assessment post-ECT treatment. In **(A)** (VIBE T1-W post-contrast sequence during portal phase in axial plane) and **(C)** (CT scan during pancreatic phase of contrast study) the arrow shows lesion. In **(B)** (VIBE T1-W post-contrast sequence during portal phase in axial plane) and **(D)** (CT scan during pancreatic phase of contrast study) the arrow shows ablated area. Qualitative assessment shows no significant differences in SI in pre- and post-treatment sequences and no significant differences in density in pre- and post-CT images.

**Figure 5:**
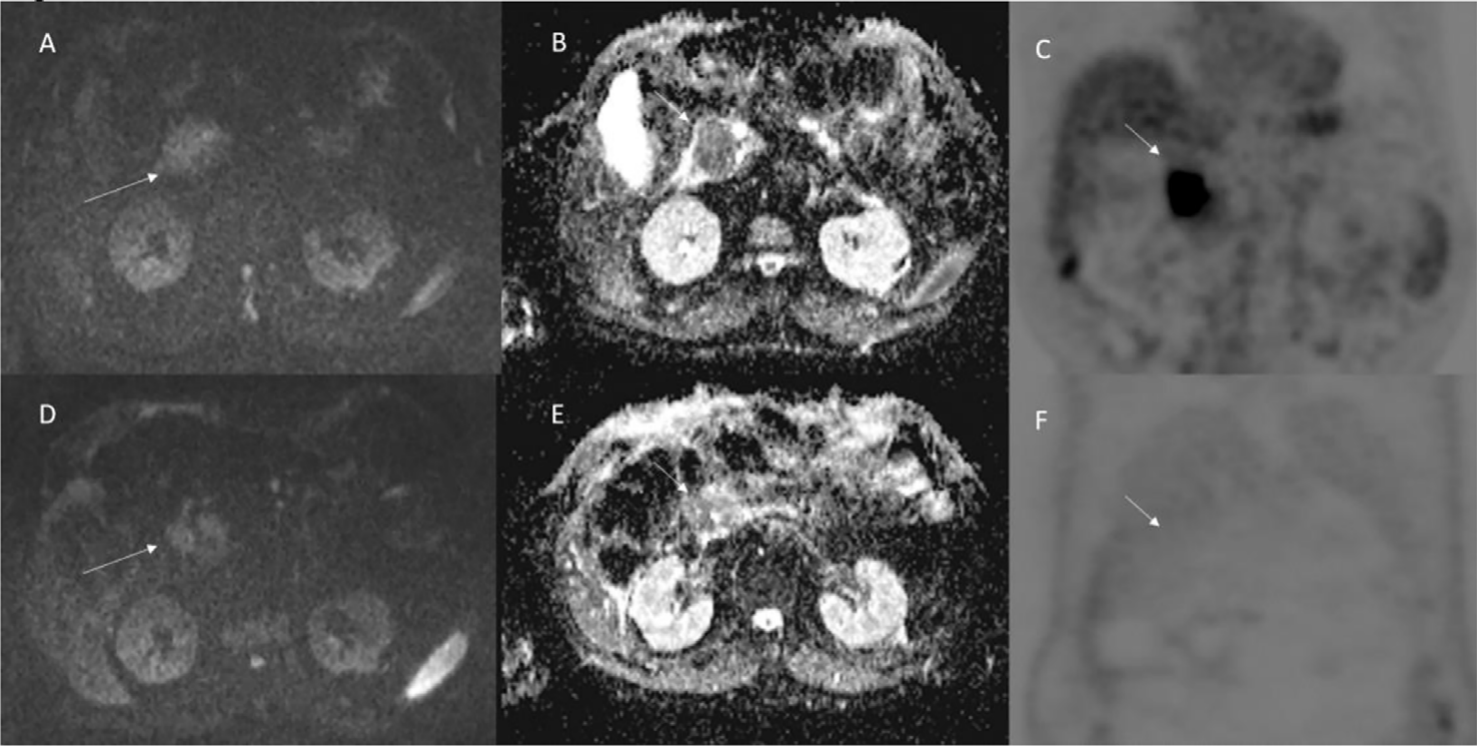
Patient 3 with head pancreatic cancer (the same of **Figure 4**). Functional (DW-MRI and PET) assessment post-ECT treatment. In **(A)** (pre treatment) and **(D)** (post treatment) (b 800 s/mm^2^) the lesion (arrow) shows restricted signal so as in ADC map (**B**: pre treatment; and **E**: post treatment) the lesion shows hypointense signal (arrow). This qualitative analysis is indicative of non-responder lesion. The PET (**C**: pre treatment; and **F**: post treatment) assessment shows (arrow) a responder lesion.

Vroomen et al. (14) assessed specific imaging features after IRE for LAPC with contrast-enhanced (ce) MRI and ce-CT, and to explore the correlation of these features with the development of recurrence. They assessed pre and post IRE, for MRI, the Signal Intensity (SI) on T2-Weigthed sequences, on T1-Weigthed sequences (before and after ce, during arterial and venous phase), on DWI and on ADC map; and for CT attenuation in the arterial and portal venous phase. They found that the most remarkable signal alterations after IRE were shown by DWI-b800 and ceMRI. According to Vroomen et al., these features may be useful to establish technical success and predict treatment outcome. Granata et al. (12, 13) assessed morphological (**Figures 3** and **4**) and functional (**Figure 5**) diagnostic parameters to evaluate the efficacy of ECT (61, 78–81). The researchers showed that RECIST criteria were not able to discriminate partial, complete, or incomplete response after treatment, conversely using functional parameters, obtained with PET and MRI, it is possible.

## Conclusions

Although new chemotherapeutic schemes have been introduced, advanced pancreatic cancers still correlate with a poor long-term outcome. Local ablative therapies are used in some dedicated cancer centers in patients with LAPC. The assessment of a pancreatic cancer after neoadjuvant treatment is particularly complicated and the condition becomes more difficult when evaluating the effectiveness of ablative therapies, considering that RECIST criteria were not appropriate to assess the treatment. When considering therapy effects, imaging-derived parameters are sometimes complicated to understand, since they depend on anatomic location, on relations between specific tissue characteristics and the mechanism of action of therapy, and on the used techniques. In this setting, imaging features are correlated to the type and method of therapy delivery, the timing of treatment, and the imaging technique being used to observe the effects. A “qualitative assessment” based only human eyes should cause misdiagnosis. A quantitative evaluation based on perfusion evaluation or metabolic analysis allows a more objective reassessment and a more correct stratification of patients in responders and non-responders to treatment.

## Data Availability Statement

The original contributions presented in the study are included in the article, further inquiries can be directed to the corresponding author.

## Author Contributions

All authors listed have made a substantial, direct, and intellectual contribution to the work and approved it for publication.

## Conflict of Interest

The authors declare that the research was conducted in the absence of any commercial or financial relationships that could be construed as a potential conflict of interest.

## Abbreviations

ADC, apparent diffusion coefficient; BLM, bleomycin; CT, computed tomography; DCE-MR, dynamic contrast-enhanced MRI; D, diffusion coefficient; Dp, pseudo-diffusion coefficient; DWI, diffusion weigthed imaging; DKI, diffusion kurtosis imaging; ECT, electrochemotherapy; EGT, electrogenetransfer; fp, perfusion fraction; IRE, irreversible electroporation; IVIM, intravoxel incoherent motion; K, kurtosis coefﬁcient; OS, overall survival; LAPC, locally advanced pancreatic cancer; MD, mean diffusivity; MRI, magnetic resonance imaging; MWA, microwave ablation; PR, partial response; PD, progressive disease; PET, position emission tomography; QoL, quality of life; RFA, radiofrequency ablation; RECIST, Response Evaluation Criteria in Solid Tumors; SD, stable diseases.
